# Mycoviroids: Fungi as Hosts and Vectors of Viroids

**DOI:** 10.3390/cells11081335

**Published:** 2022-04-14

**Authors:** Liying Sun, Ahmed Hadidi

**Affiliations:** 1 State Key Laboratory of Crop Stress Biology for Arid Areas, College of Plant Protection, Northwest A&F University, Xianyang 712100, China; sunliying@nwafu.edu.cn; 2 U.S. Department of Agriculture, Agricultural Research Service, Beltsville, MD 20705, USA

Viroids were discovered by the American plant pathologist Theodor O. Diener in 1971 [1,2] and their discovery represents the third major extension in the history of biospheres, following the discoveries of “subvisible” microorganisms by the Dutch microbiologist Antonie van Leeuwenhoek in 1675 and of the “submicroscopic” viruses by the Russian botanist Dmitri I. Ivanovsky in 1892 [3]. Viroids are infectious, self-replicating, very small single-stranded, covalently closed circular RNA molecules, with a high degree of internal base pairing, which replicate through RNA-RNA transcription [1,2]. They are the smallest known infectious RNAs with a chain length from 234 nucleotides [4] to 401 nucleotides [5]. Unlike viruses, viroids are naked, do not encode proteins, and use pre-existing host factors, host cell RNA polymerase and processing enzymes for replication and pathogenesis [1,2]. Viroids are divided into two families: *Pospiviroidae* and *Avsunviroidae* including eight genera and thirty-three species [6]. Members of the family *Pospiviroidae* replicate in nuclei and those of the family *Avsunviroidae* replicate in the chloroplasts and possess a hammerhead ribozyme in their sequencing, allowing self-cleavage during their replication.

Viroids are known to naturally infect a wide range of plants, including vegetable and field crops, fruit trees and grapevine, ornamental crops, and palm trees [7,8]. Viroids are transmitted by vegetative propagation of infected hosts, by seed, pollen, and vectors [9]. Previously, viroids were limited to plant hosts; however, recently, fungi were experimentally shown to be hosts of viroids. The term “mycoviroids” (fungal viroids) was coined by the authors earlier this year, 2022, to denote viroids that have the ability to infect and replicate in susceptible fungi [9]. This editorial discusses the current state of our knowledge on mycoviroids.

The extension of viroid host range was first reported in 2011; the avocado sunblotch viroid (ASBVd), a member of the family *Avsunviroidae*, was found to be successfully inoculated and replicated in the eukaryotic unicellular baker yeast fungus *Saccharomyces cerevisiae*, a non-plant host [10]. It was demonstrated that the plus and minus viroid RNAs were able to self-cleave and replicate in the yeast cell. This is the first report of a viroid infection and replication in fungus to provide possible extension of viroid host range from plants to different organisms. Six years later, it was also shown that potato spindle tuber viroid (PSTVd), a member of the family *Pospiviroidae*, was able to replicate in *Saccharomyces cerevisiae* [11].

Fungi are substantially different from plants. They do not have chlorophyll, and their cell walls are not made of cellulose as in the case of true plants. The majority of fungal species produce spores, which are the reproductive structures that aid in dispersal and survival. The fungus kingdom, separately from other eukaryotic kingdoms, consists of seven phyla [12], two of which, the *Ascomycota* and the *Basidiomycota*, are members of the subkingdom Dikarya; this subkingdom is characterized by septate hyphae and includes mushrooms, food-spoilage molds, the majority of plant pathogenic fungi, and beer, wine and bread yeasts. Plant pathogenic fungi belonging to class *Ascomycetes* (phyla Ascomycota) form spores in sac-like structures (asci), and class *Basidiomycetes* (phyla Basidiomycota) bear spores on club-shaped end cells called basidia. Similar to other eukaryotic organisms, plant pathogenic fungi host many viruses or virus-like RNAs, termed fungal viruses or mycoviruses [13,14,15,16].

Recently, a study has shown that the plant pathogenic fungi were able to support viroids replication [17]. Monomeric full-length RNA transcripts of seven viroid cDNA clones were artificially transfected into spheroplasts of three plant-pathogenic ascomycetes filamentous fungi, *Cryphonectria parasitica*, *Valsa mali*, and *Fusarium graminearum*, which are the causal agents of chestnut blight, apple tree canker, and wheat/barley head blight and maize ear rot diseases, respectively. The tested viroids were: ASBVd, apple scar skin viroid (ASSVd), chrysanthemum stunt viroid (CSVd), hop stunt viroid (HSVd), iresine viroid-1 (IrVd-1), peach latent mosaic viroid (PLMVd), and PSTVd. ASBVd and PLMVd are members of the family *Avsunviroidae*, whereas the other viroids are members of the family *Pospiviroidae.* ASSVd, CSVd, PLMVd and PSTVd, initially replicated in the inoculated fungal hosts, then they were eliminated after successively sub-cultured; however, HSVd, IrVd-1 and ASBVd consistently replicated in at least one of those fungi [17]. HSVd replicated in the three fungi, while ASBVd replicated in *C. parasitica* and *V. mali*, and IrVd-1 only replicated in *C. parasitica*. Most viroid infections were asymptomatic in the fungi, however, HSVd infection significantly reduced the growth and virulence of *V. mali.* [17]. This study showed that in plant pathogenic ascomycetes, inoculated viroids were transmitted horizontally through hyphal fusion and vertically through conidial transfer, further indicating the ability of this class of fungi to support viroid replication. Importantly, when HSVd-infected *F. graminearum* was inoculated to *Nicotiana benthamiana*, the plants became systemically infected with the viroid seven days later. Conversely, when viroid-free *F. graminearum* was inoculated to HSVd-infected *N. benthamiana*, the fungus acquired HSVd from plants and became viroid-infected, as shown by re-isolating the fungus from plants [17]. This two-way horizontal transfer of viroid between plant and fungus sheds light on potential new pathways of viroid transmission in the field. Notably, such a bidirectional transfer between plants and pathogenic fungi has also been demonstrated in plants with the following plant viruses: cucumber mosaic virus, tobacco mosaic virus, and fungal virus, Cryphonectria hypovirus 1 [18,19].

Further studies have shown that HSVd is able to induce epigenetic alterations through a mechanism of noncanonical RNA-directed DNA methylation. In HSVd-infected plants, high accumulation of rRNA precursors was observed, correlating with decreased DNA methylation in the promoter region of the rRNA genes [20]. HSVd was found to functionally subvert histone-deacetylase 6 (HDA6), promoting epigenetic changes in host rRNA genes during HSVd pathogenesis [21]. A lack of HDA6 activity was reported to be associated with spurious RNA polymerase II transcription of nonconventional rDNA templates (usually transcribed by RNA polymerase I) [22]. Excessive accumulation of pre-rRNAs and small RNAs derived from ribosomal transcripts was reported in HSVd-infected plants, indicating an unusual transcriptional environment [20,23,24]. Therefore, recruitment of HDA6 by HSVd may favor the transcription of noncanonical templates, thereby improving HSVd replication in infected cells.

Along with viroids and viruses, plants also host a number of parasitic organisms, including fungi, oomycetes, bacteria, and phytoplasmas; these colonizing microbes interact with each other and with the host plant. Plants have been proposed to serve as reservoirs and vectors for viruses/viroid in the environment. Figure 1 shows a model of viroid replication and transmission between plants and fungi.

Current knowledge of RNA virus lineages suggests that widespread horizontal virus transfers between diverse hosts have occurred in the past, and these contribute greatly to RNA virus evolution. To enhance their compatibility with diverse hosts and counteract or evade host defense responses, the viral genomes often mutate once they invade a novel host. The genomes of viroid progeny produced by HSVd and ASBVd after infection of *F. graminearum* and *C. parasitica*, respectively, showed nucleotide substitution [25]. Moreover, sequencing of the nucleotide sequence junction of the circularized plus HSVd accumulated in *F. graminearum* (determined by inverse RT-PCR) provided additional supporting evidence for viroid replication and adaptation in fungi and suggested that evolution of viroid genomes during replication in fungi is possible [25].

To date, no natural infections of fungi with viroid or viroid-like RNAs have been discovered. However, an increasing amount of evidence supports the view that fungi, as a eukaryotic organism, are able to host viroid replication. Interestingly, HSVd replication in *Valsa mali* significantly reduced the growth and virulence of the fungus. Thus, this mycoviroid may be exploited as a biocontrol agent to control plant pathogenic fungi. It is predicted in the near future that research on mycoviroids will be extended to other major taxa of fungi, unculturable biotrophic fungi and oomycetes, as well as to viroid species (and variants) of the two families *Avsunviroidae* and *Pospiviroidae.*

## Figures and Tables

**Figure 1:**
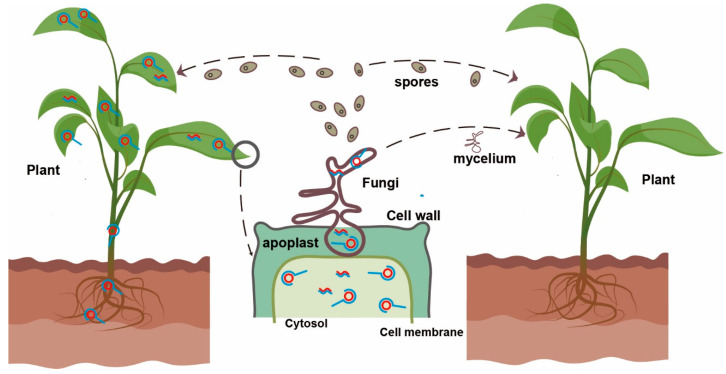
A model of viroid replication and transmission between plants and fungi. As a natural host of viroid and fungi, plants support viroid replication (shown for the plant on the left); viroid rolling-circle replication in plant and fungal cells is indicated by red and blue lines. Fungus, a potential viroid host, coincidentally infects the plant carrying the viroid. Viroid-fungus-plant interaction occurs in the apoplastic space of plants; viroid can then be acquired by the co-infected fungus and spread by the mycelium or by fungal spores. Replication in the fungal cells is also possible if the host is suitable.
